# Fine-Tuning Genetic Circuits via Host Context and RBS Modulation

**DOI:** 10.1021/acssynbio.4c00551

**Published:** 2025-01-04

**Authors:** Dennis Tin Chat Chan, Lena Winter, Johan Bjerg, Stina Krsmanovic, Geoff S. Baldwin, Hans C. Bernstein

**Affiliations:** † Faculty of Biosciences, Fisheries and Economics, UiT—The Arctic University of Norway, 9019 Tromsø, Norway; ‡ The Arctic Centre for Sustainable Energy, UiT—The Arctic University of Norway, 9019 Tromsø, Norway; § Department of Life Sciences, Imperial College London, South Kensington, London SW7 2AZ, U.K.; ∥ Imperial College Centre for Synthetic Biology, Imperial College London, South Kensington, London SW7 2AZ, U.K.

**Keywords:** synthetic biology, biodesign, chassis effect, genetic circuit, context dependence, broad-host-range

## Abstract

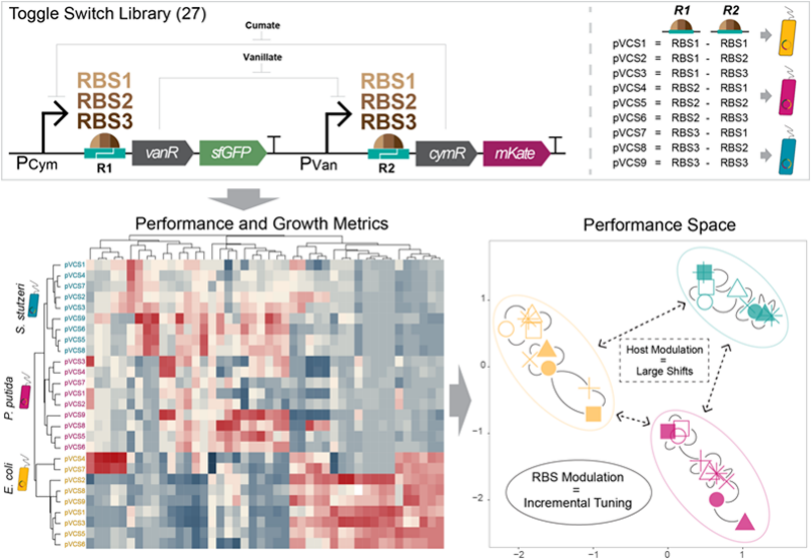

The choice of organism to host a genetic circuit, the chassis, is often defaulted to model organisms due to their amenability. The chassis-design space has therefore remained underexplored as an engineering variable. In this work, we explored the design space of a genetic toggle switch through variations in nine ribosome binding site compositions and three host contexts, creating 27 circuit variants. Characterization of performance metrics in terms of toggle switch output and host growth dynamics unveils a spectrum of performance profiles from our circuit library. We find that changes in host context cause large shifts in overall performance, while modulating ribosome binding sites leads to more incremental changes. We find that a combined ribosome binding site and host context modulation approach can be used to fine-tune the properties of a toggle switch according to user-defined specifications, such as toward greater signaling strength, inducer sensitivity, or both. Other auxiliary properties, such as inducer tolerance, are also exclusively accessed through changes in the host context. We demonstrate here that exploration of the chassis-design space can offer significant value, reconceptualizing the chassis organism as an important part in the synthetic biologist′s toolbox with important implications for the field of synthetic biology.

## Introduction

Genetic circuits have emerged as powerful tools for engineering cellular behavior,^1^ thus opening new opportunities for biotechnology to address pressing issues such as disease mitigation,^2^ climate change,^3^ and food security.^4^ Realizing these promises requires synthetic biologists to enhance the reliability and scope with which biological systems can be engineered to achieve the desired performance characteristics. The systematic design-build-test (DBT) engineering framework and implementation of standardization^5^ infrastructure within various aspects of synthetic biology has significantly accelerated advancements in this field. In the engineering of genetic circuitry, the DBT cycle is typically implemented through two primary approaches: rational forward engineering and combinatorial engineering. Forward engineering involves designing high-level constructs based on available and characterized parts, relying heavily on existing knowledge of these basal components and their interactions.^6^ Meanwhile, combinatorial engineering approaches attempt to exhaust large design spaces and, in some cases, all possible combinations of basal parts followed by downstream screening for desired performances.^7^ Contemporary biodesign often integrates these two approaches, using forward engineering to establish the core logic and structure of genetic circuits, while combinatorial methods are employed to fine-tune performance specifications through modular component assembly.^6^ This hybrid strategy maximizes the efficiency and efficacy of genetic circuit design.^8^

The performance and predictability of synthetic biological devices are influenced by the host-context in which they operate.^9−12^ Interactions between the host cell, i.e., “chassis”, and heterologous circuit machinery is complex and necessitates careful consideration to effectively navigate and exploit the genetic circuit performance space to attain desired attributes.^13^ Addressing these challenges requires precise tuning of gene expression within synthetic circuits, which has previously been achieved by modulating regulatory elements, or “parts”—in a combinatorial manner. This includes elements such as promoters,^14,15^ copy numbers,^16,17^ terminators,^18^ and ribosome binding sites (RBSs)^19,20^ designed in various circuit topologies.^21,22^ Contemporary combinatorial engineering efforts have successfully enhanced yields of microbial cell factories^16,17,23^ and improved fold-induction of biosensing devices^24,25^ by perturbing genetic components. Tuning gene expression through RBS modulation has been a particularly popular engineering strategy for multiple reasons.^26−28^ For example, the relatively short length of RBSs, which often includes spacer regions and upstream 5′-UTR components, constrains the design space to a manageable size and cost-efficient size.^29^ Still, a single nucleotide change within an RBS can lead to significant differences in translational strengths^28,30^ meaning a wide spectrum of gene expression levels can be achieved. RBSs and ribosome structures are also highly conserved across prokaryotes, thereby posing a lower risk of unspecific interactions, compared to promoter parts which are susceptible to cross-talk with transcription factors.^19^ Lastly, the development of tools such as the RBS calculator by Salis to predict translation initiation rates from RBS sequence alone has further improved the efficiency of RBS modulation.^31−33^

While significant progress has been made in manipulating genetic elements, the “chassis-design space” remains relatively underexplored.^13,34,35^ The choice of chassis to host engineered genetic circuits usually defaults to a genetically tractable model organism (e.g., *Escherichia coli*) despite the model organism not necessarily being the most optimal host.^36^ This has significant implications because the choice of host can dramatically influence circuit performance.^37,38^ The same genetic circuit can assume a variety of performance specifications depending on the host context it operates within, a phenomenon known as the chassis effect.^37−39^ The chassis effect arises from the inherent coupling of heterologous circuitry to the endogenous system, for instance, in the form of resource competition^40−43^ and/or regulatory cross-talk by promiscuous transcriptional factors.^44^ The chassis effect can also be traced to differences in rate of processes such as growth-mediated dilution of circuit elements (*e.g*. mRNA and protein products). Indeed, growth-mediated dilution has been shown to alter the logic function of genetic circuits and cause other unexpected behaviors.^45,46^ The chassis effect thereby causes challenges for biodesign but also offers opportunities. While unspecific interactions and resource competition between a synthetic circuit and the host′s native genetic machinery can undermine predictability and select against circuit-bearing cells, strategic exploitation of the chassis effect can expand upon circuit performance and be used to achieve functional capabilities that are otherwise difficult to rationally engineer forth.^47,48^ Adding chassis-design spaces to a synthetic biologist’s toolbox can revolutionize microbial biodesign strategies by enabling the discovery of performance spaces beyond the constraining reliance on traditional model organisms. Taking more advantage of innate pragmatic phenotypes in nontraditional hosts that complements a designed function also serves as an efficient design strategy for synthetic biology.^49−52^ Exploration of the chassis-design space has been hindered by a lack of understanding of which biological determinants drive the chassis effect. Previous literature has shone light on this subject by tracing the chassis effect to different in bacterial host-physiology^38^ as well as differences in differential gene expression^39^ between hosts. Furthermore, many organisms are not amenable to genetic transformation for reasons such as incompatible origins of replications for plasmid-based devices, toxicity of heterologous products, debilitating growth burden imposed by the circuit, unsuitable transformation methods, and/or uncharacterized host immune systems.^53,54^

In this study, we explored the design space of a genetic toggle switch circuit spanning nine combinations of RBS variants across three host contexts (*E. coli* DH5α, *Pseudomonas putida* KT2440, and *Stutzerimonas stutzeri* CCUG11256), creating a library of unique toggle switch variants. We systematically demonstrate the chassis effect within this library, identifying key principles for the combinatorial use of RBS and chassis contexts to explore a tunable design space. Our findings underscore the potential of integrating RBS modulation and host context variation to shape the performance landscape of genetic circuits, providing valuable insights into the broader field of synthetic biology.

## Results

### Establishing the Design Space via Combinatorial Hosts and RBS Sequences

**Figure 1:**
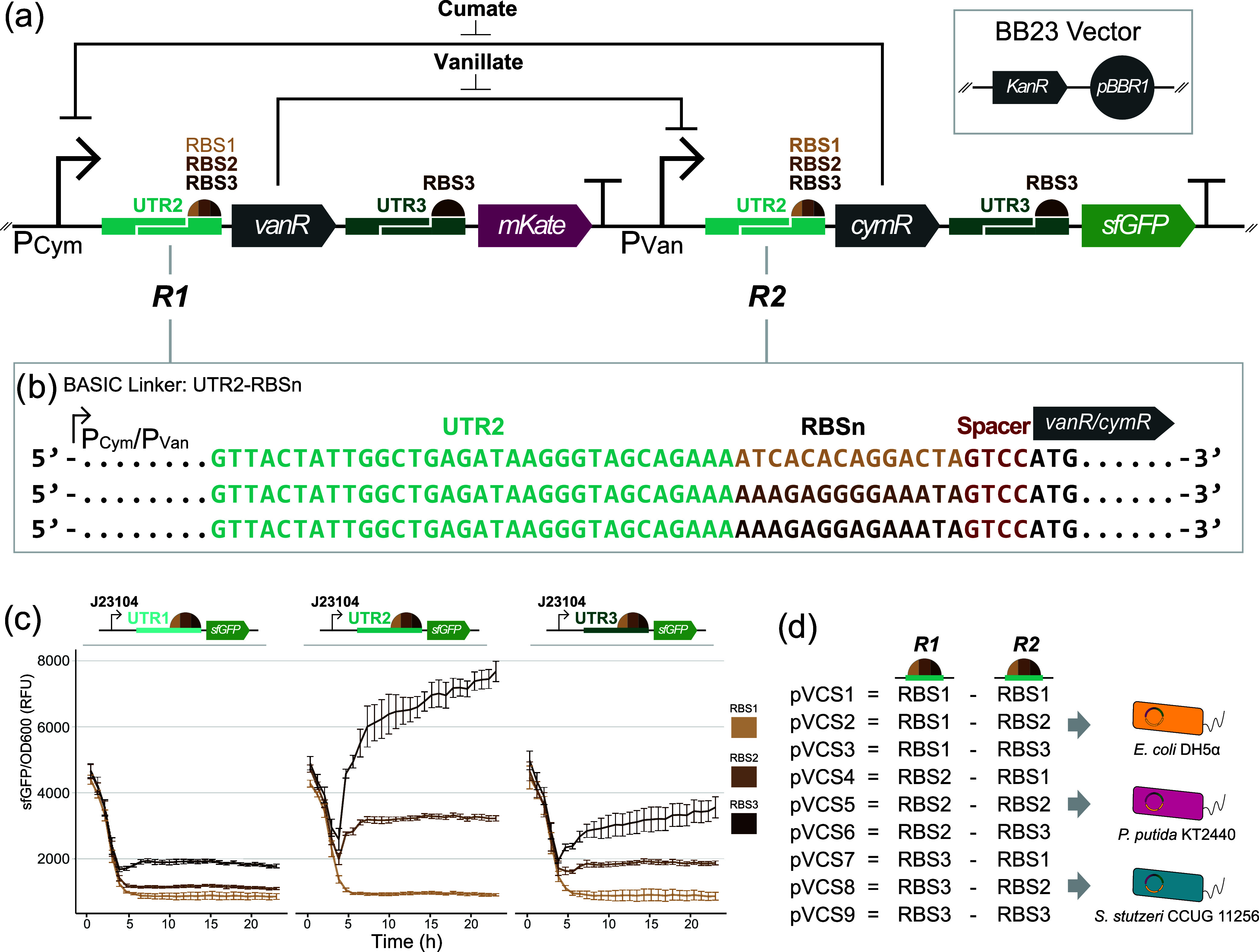
The nine pVCS toggle switches with combinatorial RBS strengths were introduced into three bacterial host contexts. (a) Core design of the cumate and vanillate inducible toggle switches, varying in the combination of RBS parts upstream of genes encoding for the two transcription factors (site *R1* and *R2*). Toggle switches were cloned into the BB23 vector with the *KanR* selection marker and pBBR1 origin of replication, yielding the pVCS plasmid series. (b) Besides the RBS parts regulating for the transcription factors, the intergenic context was held constant across designs via the BASIC assembly linker, with each RBS being preceded by the same 5′-UTR sequence (UTR2) and downstream spacer region. RBS strength regulating for reporter protein CDSs was preceded by UTR3. (c) Preliminary fluorescence assay of nine constitutive reporter circuits spanning three BASIC RBSs and three 5′-UTR regions in *E. coli* DH5α. Fluorescence output is normalized against OD600. J23104 is a constitutive Anderson promoter. Error bars show standard deviation, *n* = 4. (d) The pVCS series of nine toggle switches were successfully introduced into three host contexts, *E. coli* DH5α, *P. putida* KT2440, and *S. stutzeri* CCUG 11256.

We identified a unique performance landscape from three bacterial hosts operating a suite of genetic toggle switches constrained to the same design space defined by combinatorial pairings of RBS parts. We constructed the pVCS plasmids, a series of nine toggle switches with modulated combinations of RBS strengths regulating the translation of the two genes coding for repressive transcriptional factors (Figure 1a). The pVCS series was assembled using the DNA-BOT^55^ platform via automated BASIC DNA assembly,^56,57^ using RBSs of known relative translational strengths (RBS1, RBS2, and RBS3, in increasing strength) incorporated into the BASIC linkers. The core design of our toggle switches draws inspiration from the canonical Gardner et al. genetic toggle switch,^58^ consisting of two antagonistic expression cassettes to create a bistable motif with each cassette being regulated by a negatively inducible promoter. Transcription from either promoter leads to production of the opposite inducible promoter’s cognate repressor protein and a unique fluorescent protein, thereby establishing a mutual inhibitory regulatory network. The addition of inducer cumate (cym) or vanillate (van) positively biases transcription from the P_Cym_ and P_Van_ promoters, respectively. Besides the RBS parts, the intergenic context was kept constant across all toggle switches (Figure 1b). This includes the 5′-UTR and spacer region upstream of the start codon for the modulated RBSs and the RBSs regulating the two genes encoding fluorescent reporters (UTR3-RBS3) (Figure 1a).

A preliminary assay with constitutive fluorescence reporter constructs verified the translational strengths of the three RBS parts (Figure 1c). The assay also reveals that upstream 5′-UTR identity can greatly impact gene expression levels, the effect of which is most prominently observed for the RBS3 linker set, with a 6-fold difference in estimated steady-state fluorescence levels between UTR1-RBS3 (1860 ± 50 RFU) and UTR2-RBS3 (7010 ± 270 RFU) circuit variants. The initial decline in normalized fluorescence output is due to the fluorescence curve lagging behind the growth curve, likely due to the maturation time of fluorescent proteins.^59^ We note that all reported RFU units are normalized by OD600, which was done to control for growth effects. Open-Source Translation Initiation Rate (OSTIR) program also infers the expected increasing translation initiation rate according to the predetermined strengths of the RBS parts under the context of the actual toggle switch designs (Supporting Figure S1). Together, these results lend power to the use of the BASIC RBS linkers as a strategy to fine-tune circuit performance. The pVCS plasmid series, using the pBBR1 origin of replication, was transformed successfully into all three host species (Figure 1d) and sequence verified, yielding a library of 27 toggle switches spanning nine RBS combinations and three host contexts. With this experimental framework, we set out to characterize the performance variability under standardized conditions and elucidate the efficacy of the two design spaces as strategies to tune circuit function.

### Variable RBS Pairings Lead to Diverse Performances across Hosts

**Figure 2:**
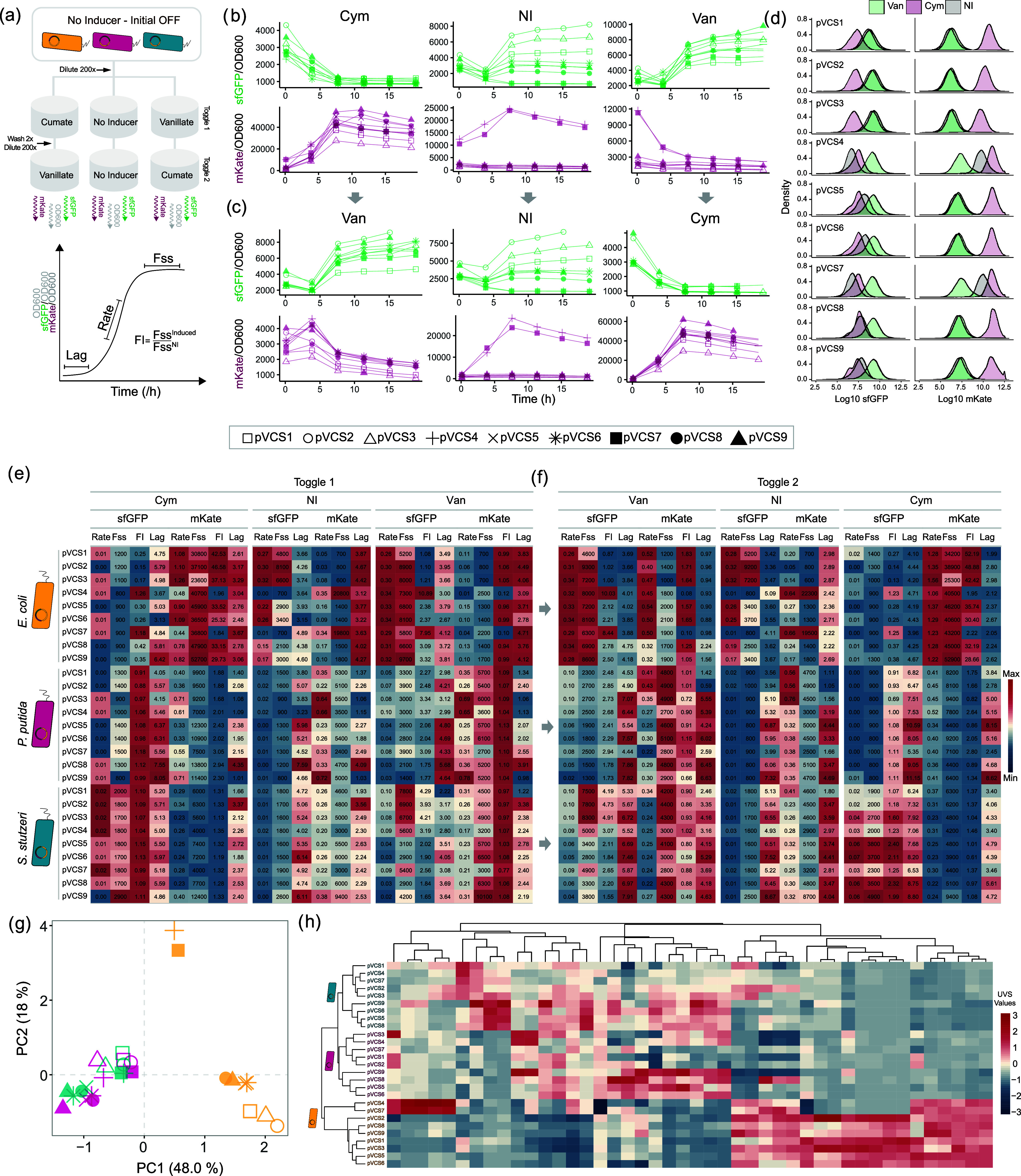
Toggle switch performance clusters by host context. (a) Schematic of toggling assay. Cells in the initial OFF state were diluted to 0.75 mM cym and van, as well as a no inducer (NI) control. To toggle, cells were washed twice before being diluted 200× to respective opposite induction states under the same concentration. The metrics Lag (lag time before fluorescence increase in units of hours), Rate (rate of exponential fluorescence increase in units of RFU/h), and *F*_ss_ (steady-state fluorescence at late phase in units of RFU) were estimated from growth normalized sfGFP and mKate fluorescence curves. Fold-induction (FI) is defined as the ratio between induced and noninduced *F*_ss_. (b) Representative normalized fluorescence dynamics across induction states for toggle switch-carrying *E. coli* cells toggled from initial OFF and (c) toggled to opposite induction state. (d) sfGFP and mKate fluorescence intensity distribution of *E. coli* cell populations. Performance metrics across the 27 toggle switch variants from (e) toggle 1 and (f) toggle 2 across induction state for both outputs. Color scale indicates the maximum and minimum value relative to each column. (g) Principal component analysis plot of all quantified toggle assay metrics. (h) Euclidean distance-based hierarchical clustering of circuit contexts and toggle switch performance metrics. UVS = unit-variance scaled. *n* = 4.

Comparison of performance profiles among toggle switch variants reveals that the host context has a more significant influence than the RBS context on device performance. Specifically, variations in host context lead to more substantial shifts in the overall performance profile, whereas changes in the RBS context result in more incremental adjustments. Performance metrics were derived from the fluorescent response dynamics of the circuit variants across induction states in a toggling assay (Figure 2a). These measurements include measurements of lag time (Lag, in units of h), rate of exponential fluorescence increase (Rate, RFU/h), and steady-state fluorescence output at the stationary phase (*F*_ss_, in units of RFU). The biological interpretations of these metrics vary according to the state of induction. For instance, in the absence of inducer, the Rate and *F*_ss_ metrics for sfGFP and mKate represent expression leakage or baseline output. Conversely, sfGFP output in the presence of cym (or mKate in the presence of van) indicates expression leakage from the P_Van_ promoter despite supposed VanR repression. We also define fold-induction (FI) as the ratio between induced and noninduced *F*_ss_, which informs of the responsive range of the toggle switch.

In toggle 1, cells were induced from an initial OFF state to van-ON or cym-ON while also maintaining a control culture with no inducer (NI). A second phase of the circuit, toggle 2, was then activated with the alternate induction state after dilution and subsequent growth to stationary phase (Figure 2b,c and Supporting Figure S2). Examining the fluorescence responses at individual cell level via flow cytometry verified a uniform population response from all 27 toggle switch variants in stationary phase (Figure 2d and Supporting Figure S2). Comparing the quantified performance metrics reveals a diverse range of performance profiles, demonstrating how varying the RBS and host context can grant access to a wider set of performance specifications (Figure 2e). Switches operating within the context of *E. coli* exhibited an overall stronger sfGFP and mKate response, as indicated by the higher Rate and *F*_ss_ values. For instance, cym-induced mKate *F*_ss_ levels range from 23,600 ± 1500 RFU (pVCS3) to 52,700 ± 1200 RFU (pVCS9) in *E. coli*, while the highest mKate *F*_ss_ values achieved in *P. putida* and *S. stutzeri* were 13,800 ± 500 (pVCS8) and 12,400 ± 170 RFU (pVCS9), respectively, 2-fold lower than the lowest output attained by pVCS3 in *E. coli*. This suggests that *E. coli* provides an environment conducive to a higher level of fluorescent protein accumulation.

A high fold-induction (induced signal-to-baseline ratio) is usually desired in genetic devices such as chemical event detectors.^37,60^ When implementing this current toggle switch design into *E. coli*, the circuits gain access to much higher fold-induction response upon cym induction (measured via mKate fluorescence reporting) as compared to van measured by sfGFP. While *E. coli* cells exhibit some of the highest sfGFP *F*_ss_ values among all circuits, they were all accommodated with high baseline, resulting in overall similar responsiveness across hosts (average sfGFP_Van_ FI value for all 27 strains is 3.0 ± 2.1). Accessing circuit variants that operate similarly in function but at different amplitudes can be beneficial, especially when the baseline signal of some circuits saturates the measurable range of a fluorescent reader or when background noise in samples is high. Cells toggled between induction states showed the expected inversion of fluorescence response according to their design (Figure 2f), albeit with some devices toggled from van-ON to cym-ON exhibiting a slightly attenuated response in *P. putida* and *S. stutzeri*. This response attenuation appears to be dependent on past induction states, as cells toggled from cym-On to van-ON demonstrate similar performance as cells toggled from initial OFF across contexts and only minute concentrations should remain after washing and dilution. Principal Component Analysis (PCA) of performance profiles data set clusters toggle switches in *P. putida* and *S. stutzeri* into their own cluster with almost equal dissimilarity to *E. coli* (Figure 2g). The spread of toggle switches within each host cluster illustrates the incremental adjustments that occur when varying RBS combinations, highlighting the fine-tuning capability of varying RBS parts. Notably, *E. coli* pVCS4 and pVCS7 form their own distinct cluster, diverging in behavior even from other *E. coli* toggle switches. Indeed, closer inspection of pVCS4 and pVCS7 metrics reveals a high mKate and low sfGFP baseline, suggesting the native state of these designs is more biased toward expression from the P_Van_ promoter, likely due to their unique RBS combination. This opposite base state appears to be the cause of their opposite behavior. For instance, the mKate_Cym_ FI values of these two switches are 2.0 ± 0.1 and 1.8 ± 0.1, respectively, manifolds lower than the FI values of the other seven *E. coli* switches, which range from 25.3 ± 1.0 to 46.6 ± 2.7. Meanwhile, pVCS4 and pVCS7 achieve the highest recorded sfGFP_Van_ fold-induction values out of any device (10.9 ± 0.7 and 8.0 ± 0.5, respectively).

The different hosts revealed preferential base-level operation of the toggle switch under noninduced state. Toggle switches in *P. putida* and *S. stutzeri* demonstrate higher average mKate *F*_ss_ values (mKate_NI_*F*_ss_ 4600 ± 1000 and 5200 ± 1900 RFU, respectively) than those in *E. coli* (average 1100 ± 500 RFU among circuits, excluding pVCS4 and pVCS7). The opposite is observed for the sfGFP baseline output, with toggle switches in *E. coli* exhibiting higher average sfGFP_NI_*F*_ss_ values. These observations suggest that the host environments greatly affect the native toggled state of the device, which in turn affects circuit performance and underscores the substantial impact of host context on genetic circuit behavior.

Hierarchical clustering of performance profiles reveals that toggle switches separate according to the three host contexts, branching *P. putida* and *S. stutzeri* into their own clusters (Figure 2h). Interestingly, within the *P. putida* and *S. stutzeri* cluster, specific combinations of RBS strengths found in pVCS1, pVCS2, pVCS3, pVCS4, and pVCS7 clusters separately from the other four circuits. A similar clustering within *P. putida* and *S. stutzeri* clusters is observed in Figure 2g, indicating in cases of little phylogenetic difference between hosts that similarity in RBS combinations does lead to similar performance. Overall, our results suggest that changes in host context cause significant shifts in overall toggle switch performance, whereas modifications in RBS context typically result in minor changes. However, in certain instances, such as with the pVCS4 and pVCS7 variants in *E. coli*, RBS changes can also greatly alter circuit performance, highlighting the importance of exploring a wide design space.

### Species-Specific Growth Physiology Imposes Limits on Toggle Switch Performances

**Figure 3:**
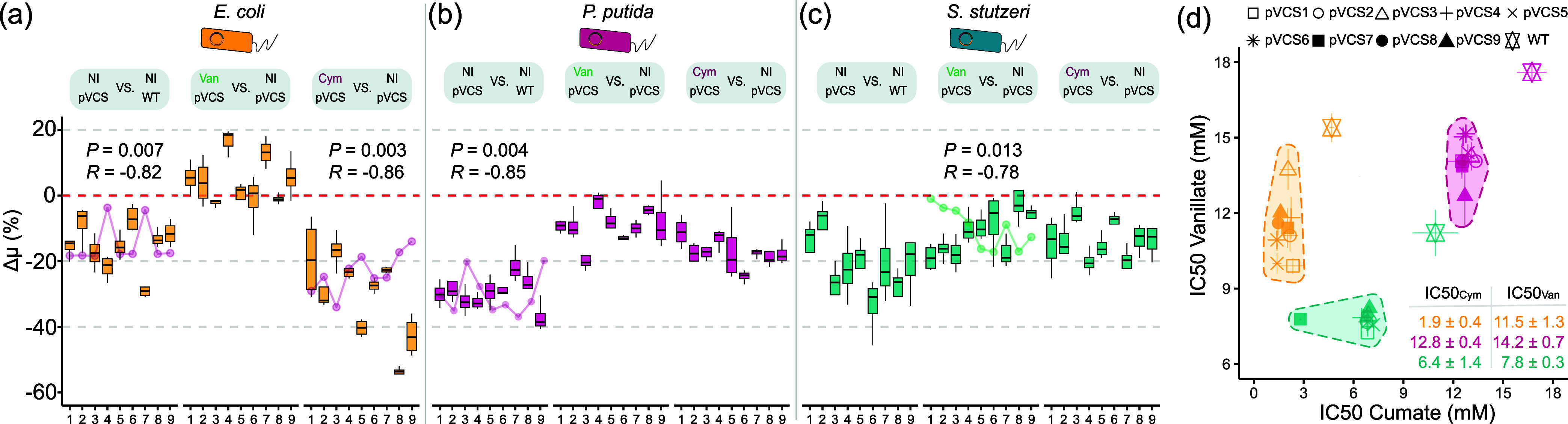
Coupling between the circuit function and chassis growth leads to operational limits of toggle switches. Growth burden quantified through the relative percentage difference in growth rate (Δμ) between noninduced (NI) plasmid-bearing strains and WT, van-induced against NI, and cym-induced and (NI) strains for (a) *E. coli*, (b) *P. putida* and (c) *S. stutzeri*. The Δμ metric reflects the percentage difference in growth rate between two conditions (one control and one treatment), with a negative value representing a decrease in growth rate and vice versa. In comparisons where growth burden and sfGFP/mKate output metric (product of Rate and *F*_ss_) was significantly correlated, the associated *P*-value (*P*) and Pearsons’s correlation coefficient (*R*) are shown. (d) Half-minimum inhibitory concentration (IC_50_) of vanillate and cumate for each strain. Shaded areas are clustered by host, excluding WT. Table inset shows average IC_50_ values within each inducer-host group. Error bars show standard deviation, *n* = 4.

Introduction of a genetic circuit into a host environment couples the exogenous circuit to the host′s cellular machinery, causing a reallocation of resources originally dedicated to growth and cell maintenance.^40,61,62^ Circuit performance is therefore constrained by the unique growth physiology of each host and physiological responses to the toggle switch.^38^ To elucidate this host-device interplay on growth, we determined the growth burden caused by the toggle switch operation. Growth burden was quantified through the Δμ metric, defined as the relative percentage difference in specific growth rate (μ) between two conditions. Growth dynamics of the toggle assay were measured simultaneously and by comparing plasmid-bearing strains in baseline state against their wildtype (WT) genotype counterpart (Figure 3a and Supporting Figure S3). The results show that maintaining the toggle switch consistently imposes a reduction of growth across all hosts but to various degrees.

The most severe growth burden associated with the introduction of the pVCS plasmid was observed among *P. putida* strains with plasmid-bearing cells experiencing on average −29.3 ± 4.4% lower growth rate than their WT counterpart. *E. coli* and *S. stutzeri* cells experienced on average a more moderate −15.6 ± 6.9 and −22.2 ± 7.4% lower growth rate compared to their WT counterparts. The pBBR1 origin of replication has been reported as a low-copy number plasmid in *E. coli*, but previous studies has shown that plasmid copy number is subject to the chassis effect and that *P. putida* can maintain a 10-fold higher plasmid copy number of a pBBR1 plasmid compared to *E.coli*,^38,63^ which could explain the higher degree of growth burden. Further induction of the switches exaggerates growth burden, with toggle switches operating from *E. coli* experiencing the most drastic growth inhibition upon cumate induction, but the degree of growth burden ranges widely from −16.8 ± 5.4% (pVCS3) to −53.0 ± 1.8% (pVCS8). Pearson correlation analysis reveals significant negative correlation between mKate_Cym_ output and Δμ metric in *E. coli* variants induced with cym (*P*-value = 0.007, *R* = −0.82). This suggests the degree of growth burden correlates with output, which is in turn tunable with RBS. The same negative significant trend is also observed for van-induced *S. stutzeri* variants and sfGFP_Van_ output and noninduced mKate output in *E. coli* and *P. putida*. Stronger output can thereby lead to greater growth burden, which has major implications toward tuning genetic circuits within the bounds of species-specific growth constraints. This result also highlights the importance of optimizing not only for performance but also for growth when it comes to working with living systems. The positive Δμ values for certain *E. coli* strains in the van-induced against noninduced comparison group implies that the presence of vanillic acid provides some form of growth benefit, but this seems highly unlikely given that previous reports have highlighted the potential antimicrobial properties of vanillic acid^64^ and no other similar positive growth difference was observed for *P. putida* and *S. stutzeri*. We have previously demonstrated that differences in growth physiology significantly correlated with differences in performance,^38^ which we once again observe when comparing the differential performance of all 27 toggle switches and their differential growth physiologies through Procrustes Superimposition analysis, solidifying our previous findings (Supporting Figure S4).

Exploring both the host and RBS design space allows the discovery of a suitable version of a circuit that balances growth and performance. The results shown thus far establish an interplay between device and growth physiology, which is complex and depends on the specific choices of bacterial host species, RBS combinations, and user-defined operation of the toggle switch. Our results show clearly that toggle switches result in burdened growth and, therefore, tax the host of its cellular resources. This effect results in lower cell division rates, which in turn impose reciprocal constraint back onto circuit performance within the operational concentration limits defined by the induction kinetics of P_van_ and P_cym_, respectively. We identified these limits by determining the half-minimal inhibitory concentration (IC_50_) of each inducer module (Figure 3d). A clustering of toggle switches by their host context is again observed. *P. putida*, an established model organism known for its robustness,^65^ is the most tolerant to both inducers with some of the highest IC_50_ values, particularly for cumate. *E. coli* toggle switches exhibit the lowest tolerance to cumate, with an average IC_50Cym_ of 1.9 ± 0.4 mM. WT strains of hosts all demonstrate higher tolerance to inducers, as expected due to not being burdened with maintaining a foreign plasmid. The clustering of IC_50_ values within each inducer-host group shows minimal variation among RBS variants, highlighting the limitations of using RBS modulation to enhance the inducer tolerance. In contrast, altering the host context emerges as an effective method to enhance inducer tolerance and by extension can be used to engineer forth performance capabilities beyond those achievable through intracircuit context alone.

### The RBS Design Space Allows for Precision Tuning of Species-Specific Operation Objectives

**Figure 4:**
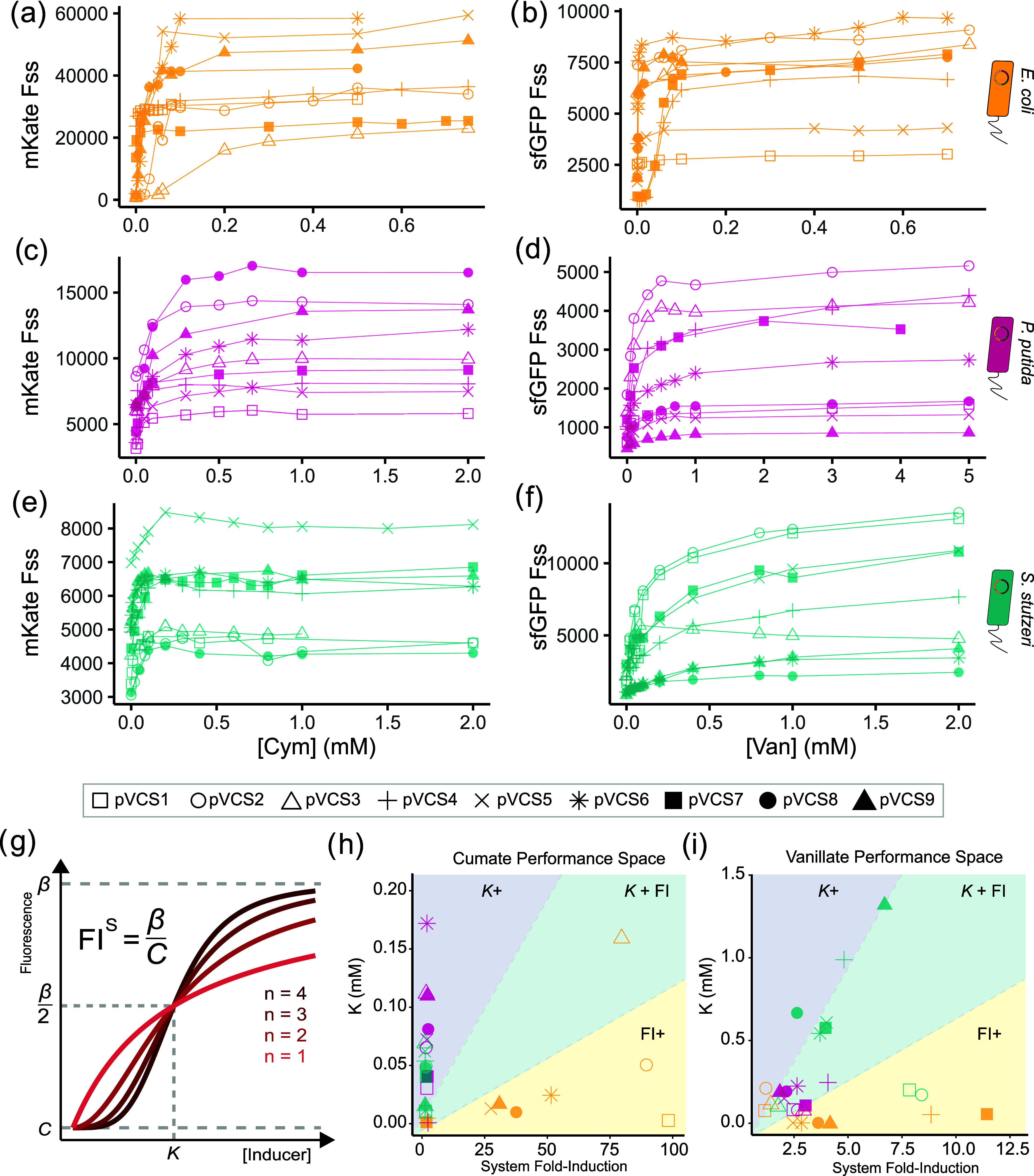
Exploration of the chassis-RBS design space reveals chassis exclusive performance spaces. Cumate and vanillate induction response curves of the pVCS toggle switches for (a, b) *E. coli*, (c, d) *P. putida*, and (e, f) *S. stutzeri*. Note differences in the axis scales. (g) Visual representation of Hill function curve with Hill coefficients (*n*) and the other three estimated parameters: *C* (fluorescence steady-state output in the absence of inducer or leakage), *K* (activation coefficient or inducer concentration in which half-maximal output is attained) and β (fluorescence steady-state at saturating inducer levels). System fold-induction (FI^S^) is the ratio of the estimated β and *C*. The sampled (h) cumate and (i) vanillate toggle switch performance parameter space, plotting activation coefficient and system fold-induction. Dashed lines arbitrarily divide regions designated as sensitivity optimized (*K*+), reporting optimized (FI^S^+) and both (*K* + FI^S^). Error bars are the standard deviation, *n* = 7.

Modulating the host context was found to greatly alter the induction kinetics of the genetic circuits, whereas varying the RBS strength is ideal for fine-tuning of performance objectives. The induction kinetics of all 27 toggle switch variants were determined to gain a comprehensive assessment of their performance across a wider range of concentration states (Figure 4a–f), yielding deeper insights into the unique performance characteristics not revealed from a single induction point. The Hill function was fitted to induction curves to estimate the system parameters β (Figure 4g), which represents the max fluorescence output at saturating inducer concentrations; the activation coefficient *K*, indicating the inducer concentration at which half-maximum output is achieved; and *n*, the Hill coefficient serving as a fitting parameter. The system fold-induction (FI^S^) is calculated as the ratio between β and *C*, where *C* represents the empirically determined fluorescence output at a 0 inducer concentration. Collectively, these performance metrics provide a detailed description of each toggle switch variant’s performance specification.

The estimated *K* values indicate that appreciable cumate and vanillate induction can occur in the micromolar concentration range, consistent with previous literature describing P_Cym_ and P_Van_ induction systems.^66−69^ A wide range of inducer sensitivities are observed. For instance, the *E. coli* toggle switches demonstrated some of the lowest *K*_Van_ values, with an average value of 0.059 ± 0.005 mM, approximately 10-fold lower than the average *K*_Van_ values for *S. stutzeri* (0.54 ± 0.16 mM), suggesting the cellular context of *E. coli* alters the toggle switch’s responsiveness to induction. Meanwhile, cumate induction appears to require lower concentrations overall, as all *K*_Cym_ values range from 0.002 ± 0.004 to 0.156 ± 0.004 mM across all variants.

The observed performance space of the toggle switch library, visualized by plotting *K* against FI^S^ (Figure 4h,i), reveals that certain performance specifications can only be accessed by varying host context. Considering cumate induction kinetics, toggle switches operating within a *P. putida* or *S. stutzeri* context achieve FI_Cym_^S^ values up to 3 at most (Figure 4h). When operating within *E. coli* however, a toggle switch with FI_Cym_^S^ value up to 98.6 can be achieved, a clear example of how varying host context rather than intragenic context can be used to tune function. These *E. coli* switches, however, show little variation in terms of *K*_Cym_ values, limiting their sensitivity and inducible range. Meanwhile, the same toggle switches functioning within *P. putida* and *S. stutzeri* hosts (with low FI_Cym_^S^) outperform *E. coli* variants in terms of *K*_Cym_ values, exhibiting a much wider range of *K*_Cym_ values. Next, considering vanillate induction kinetics (Figure 4i), *E. coli* switches again demonstrate relatively constant K_Van_ values across RBS variants and vary more in terms of fold-induction. *P. putida* switches form a tight cluster, showing little diversity in their performance compared to the more spread *S. stutzeri* group. Notably, varying the RBS composition leads to host-specific changes in performance. For instance, switches in *E. coli* only spread along the *x*-axis, while *P. putida* and *S. stutzeri* variants cluster along the *y*-axis. The spread of toggle switch variants along each host group suggests that RBS can be used to fine-tune performance, but on the other hand, this is an example of how host context can confine the performance of the genetic circuit. Overall, our results show that a combined approach of intergenic and interchassis exploration serves as a method for surveying performance spaces to gain access to circuits with more diverse performance specifications.

## Discussion

Synthetic biology is steadily advancing past its proof-of-concept stage. To fully realize its full potential, the field must work toward not only developing proof-of-concept of novel capabilities but also optimizing them for practical use. Advancing beyond the constraints imposed by working with a few preferred model organisms is an important step in this regard. In this work, we defined a combinatorial toggle switch design space based on RBS and host context and observed variable device performance within this framework. The three host contexts sampled were each associated with an optimized performance. While this optimization can come with trade-offs, it showcases the potential of modulating the chassis to tune circuit performance. We demonstrate that through combined adjustment of RBS strength and host context, the performance of a genetic toggle switch can be optimized toward high sensitivity (i.e., low *K*), high induction range (i.e., high *K*) or reporting efficiency (i.e., high FI). We thereby provide synthetic biologists with new insight into how the chassis and RBS combinatorial design space can be exploited to optimize genetic circuits to achieve desired outcomes. Furthermore, we show that certain parameters, such as increased inducer tolerance, are exclusively accessed by varying chassis contexts, further highlighting the value of broadening the available chassis-design space.

Our characterization of toggle switch performance shows that the cellular environment imposes a general floor (fully repressed output) and ceiling (fully induced output) limits upon the toggle switch as well as influencing the unbiased toggled state of the circuit (expression more toggled toward P_Van_ or P_Cym_ in the absence of no inducer). The influence of host context on circuit performance can originate from a range of host-circuit interactions. For instance, differences in minimum output could be due to the promoters recruiting RNA polymerases to different degrees of efficiency (host-specific promoter strengths),^70^ differing plasmid copy number,^38^ or promiscuous binding of the circuit’s transcriptional factors within the host genome^71^ leading to higher steady-state leakage. A cellular environment that imposes a lower turnover rate on the fluorescent proteins, more efficient folding of heterologous proteins, or higher free ribosome and/or RNA polymerase could lead to increased gene expression as well. Previous studies on broad-host-range operation of genetic toggle switches have established significant correlation between differential performance and differential growth dynamics.^38,39^ This can be rationalized by the fact that changes in growth represent a change in the physiological state of the cell including resource pool and cell-wide parameters such as transcription, translation, degradation, and dilution rates.^72^ We chose to limit our exploration of intragenic context to RBS parts only as RBS parts are relatively short (18 bp, Figure 1), meaning there is a lower chance of acquiring mutations, which was important for this study to ensure that any difference in device function stems from the host context. An investigation into how the promoter space, another modulable and impactful genetic part, would affect circuit function across host context would have garnered valuable insight. It would be particularly interesting to investigate how the performance of negatively and positively inducible promoters would behave across host context, which has implications in scaled-up applications employing two-stage fermentation strategies.^73^ It must be noted that adding an additional variable dimension would inflate the sample sizes considerably; such combinatorial explosion can, however, be handled with more powerful automation. Procrustes Superimposition analysis on our expanded sample size of 27 toggle switches reports that variants with more (dis)similar growth dynamics also exhibit more (dis)similar performance. Growth dynamics as determined here in this study can be practically measured compared to other gene expression parameters (e.g., ribosome or RNA polymerase abundance), and its significant correlation with performance thereby makes it a practical input parameter for machine learning algorithms to predict chassis effect.^74^ Integrating machine learning to predict genetic circuit performance would however require greater standardization in the empirical characterization of genetic circuits.^75,76^

The practical application of a designed microbial system requires multiple performance parameters to be optimized,^36^ and a top performer is often selected for by compromising between parameters.^7^ For instance, as we report in this work, high output levels are known to be negatively correlated to growth^77^ (Figure 3), the latter being a crucial factor that must be balanced for when working with living systems. Selecting an optimal performer must be guided by user-defined specifications and mission requirements. Considering our toggle switches as example circuits, the markedly high fold-induction of most toggle switches implemented in *E. coli* makes them the most optimal cumate sensing devices, but their low cumate tolerance limits them to cases where cumate concentration does not exceed well beyond 1.9 mM. For higher concentration detection, users will have to select a toggle switch implemented within *P. putida*, which displays higher cumate tolerance, while sacrificing reporting strength within acceptable levels. Besides fold-induction and inducer tolerance, factors such as response time, signal amplitude, and sensitivity must also be weighed and balanced to determine the optimal performer. The signal amplitude of the system must be within the detectable range of the measurement device available (suitable *C* and β). The chosen system must be sensitive enough to be able to detect the chemical in question (low enough *K*) and if titration of sample concentration is desired, a circuit with high *K* would be optimal (circuit in *P. putida* or *S. stutzeri*). On the other hand, if only the presence/absence of a given chemical is of concern, parameter *K* can be sacrificed for shorter response time (a smaller lag phase) and higher fold-induction (circuit in *E. coli*). Sampling a wider performance space increases the chances of discovering optimal performance specifications, and we have here exemplified the value of exploring the chassis-design space by demonstrating how the host context can be leveraged to gain access to performance specifications not achievable through RBS alone.

Numerous methods to minimize context dependency in hopes of increasing stability and predictability of genetic circuits have been developed. This includes tools to segregate interactions between heterologous and native components (i.e., orthogonalization) and the practice of genome reduction.^78^ Orthogonalization includes use of orthogonal ribosomes,^79,80^ orthogonal RNA polymerases^81^ and the practice of encoding circuit DNA in alternative codes only decipherable by said orthogonal machinery.^82^ A reduced genome context with only genes essential for growth in controlled laboratory conditions is believed to mitigate the risk of unspecific interactions and increased host fitness.^80^ Indeed, there is a prevalent notion that an ideal chassis organism is one with a reduced genome due to its supposed reduced context complexity.^83−85^ In contradiction to this notion, we, and others,^37,47^ have shown that there is value to be gained by instead taking advantage of the contextual diversity that resides within different chassis organisms. Our findings push the reconceptualization of the role of the chassis organism as both a valuable part of the synthetic biology toolkit and a design variable. This notion promotes the strategic utilization of the inherent diversity found in different species to optimize the genetic circuit performance. While added complexity can certainly mean increased risk of failure, this can be overcome by expanding the explored design space through high-throughput DNA assembly and screening technology, which is continuously advancing.^86^ Better yet, a merging of strategies from the two perspectives, such as applying tailored genome reduction to chassis organisms that maintains the desired innate phenotypes, can reap the benefits of both approaches. Examples of targeted genome deletion studies on both model and nonmodel organisms have already resulted in improved user-defined performance.^78,87−89^ With the steadily increasing number of organisms with pragmatic phenotypes being domesticated as biotechnology platforms,^90−94^ we envision a future where choice of host organism becomes a staple design factor in combinatorial engineering endeavors, in equal footing of promoters and RBS strengths, which will surely enhance our ability to engineer forth solutions through biology.

## Materials and Methods

### Species, Cultivation, Cloning, and Transformation

Overview of the species used in this study can be found in Supporting Table S1. Cells were cultured in LB media at 37 °C unless specified otherwise, inoculating with single streaked colonies. BB23 backbone and pVCS-carrying strains were cultivated in the presence of 50 μg/mL kanamycin while wild types were grown without. 199 μL of media was inoculated with 1 μL of overnight culture in black clear-bottom 96-well plates (Thermo Fischer, 165305) and sealed with Breath-Easy film (Sigma-Aldrich, Z380059). OD_600_, sfGFP (Ex 485/Em 515, gain 75), and mKate (Ex 585/Em 615, gain 125) fluorescence was measured continuously using a Synergy H1 plate reader (Agilent Bio-Tek, Serial Number 21031715) with continuous linear shaking (1096 cpm, 1 mm) at 9 mm read height. Working stock solutions of 1 M vanillic acid (Thermo Fisher Scientific, 10228789) stock and 1 M cumate (Sigma-Aldrich, 536663) were prepared by dissolving powder in 70% ethanol, respectively, supplemented with 150 μL of 3 M of NaOH. Cloning was performed using *E. coli* DH5α, made chemically competent, and transformed following the Inoue method.^95^*P. putida* and *S. stutzeri* were transformed via electroporation method as previously described.^38^

### Automated BASIC Assembly and DNA-BOT

pVCS plasmids were assembled in the Biopart Assembly Standard for Idempotent Cloning (BASIC)^55,56^ cloning environment. Automated BASIC assembly was performed as described in Storch et al.^55,57^ using the DNA-BOT platform with the OpenTrons2 liquid handling robot (Opentrons, 999–00111) and temperature module. BsaI-HFv2 restriction enzyme and T4 DNA ligase were purchased from New England Biolabs (R3733 and M0202L, respectively). Mag-Bind TotalPure NGS (Omega Bio-Tek, M1378–01) was used to manually purify restriction–ligation reactions as per the manufacturer’s instructions. DNA sequences for the pVCS part components can be found in Supporting Table S2. Part and assembly input files for the DNA-BOT can be found in Supporting Information S1.

### RBS Translation Initiation Rates Prediction with OSTIR

OSTIR (Open-Source Translation Initiation Rates, v1.1.2, https://github.com/barricklab/ostir) was run with default settings, using the highly conserved anti-Shine-Dalgarno sequence of *E. coli* (5′-ACCTCCTTA-3′).^96^ Briefly, OSTIR employs a thermodynamic model (ViennaRNA^97^) of bacterial translation initiation to calculate the Gibbs free energy of ribosome binding, which infers the Gibbs free energy change to a protein coding sequence’s translation initiation rate and thereby expression strength. As an input sequence, the 33 bp 5′-UTR linker region upstream of the 14 bp RBS was included. The RBS is immediately followed by a 4 bp (5′-GTCC-3′) spacer region and subsequently the gene CDS, which all begin with the start codon 5′-ATG-3′. Genes *cymR* and *vanR* have the 5′-UTR2 (5′-TGTTACTATTGGCTGAGATAAGGGTAGCAGAA-3′) sequence upstream of their RBS, while genes *sfGFP* and *mKate* have the 5′-UTR3 (5′-GTATCTCGTGGTCTGACGGTAAAATCTATTGT-3′).

### Toggle and Growth Assay and Flow Cytometry

Overnight culture grown in the absence of inducer was used to inoculate media in 96-well plates supplemented with cym, van, and no inducer condition. To toggle, cells were harvested by centrifugation at 4000 rpm for 20 min at room temperature and supernatant removed before resuspending in 200 μL of LB media, this washing step was repeated for a total of two washes. After final resuspension, 1 μL of washed cells were inoculated to 199 μL of fresh media supplemented with the opposite respective inducer. The growth difference metric Δμ was calculated using eq 2.
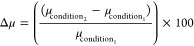
1where μ is the specific growth rate, and “condition_1” and “condition_2” denote sample conditions in terms of genotype and induction state. In R, rates of OD_600_ (specific growth rate) and normalized fluorescence curves were estimated based on a rolling regression method using the “all_easylinear” function from the growth rates (v.0.8.4, https://CRAN.R-project.org/package=growthrates) R package. Lag times and curve plateaus of OD_600_ and normalized fluorescence curves were determined using the “all_growthmodels” function, fitting the Gompertz growth model^98^ with additional lag (λ) parameter.

Flow cytometry was performed using the BD LSRFortessa Cell Analyzer (BD Sciences) equipped with an HTS autosampler (BD Sciences) measuring sfGFP signals with a 488 nm laser and 530/30 nm detector and mKate with a 561 nm laser and 610/20 nm detector. Voltages for detecting forward scatter, side scatter, sfGFP, and mKate were adjusted to 420, 270, 460, and 530, respectively. To reduce background debris, thresholds for forward and side scatter were set to 5000, while 20,000 events were recorded. Van-ON, Cym-ON, and NI cells at exponential late phase were fixed with formaldehyde to a final concentration of 1.5% and a standardized OD_600_ of 0.2.

### Induction Assays

Overnight culture grown in the absence of inducer was used to inoculate media with various concentrations of cym and van in 96-well plates. The normalized steady-state fluorescence at late growth phase (*F*_ss_) averaged over a time window of 6-12 h was used as response variable of induction curves. In R (v4.3.1), Hill coefficient (*n*), activation coefficient (*K*), and max steady-state fluorescence output (β) were estimated by fitting the Hill function (1) using nonlinear least-squares regression with the “nls” function from the stats base R package. For parameter *C*, representing basal fluorescence output at 0 inducer concentration, an empirical value was used.
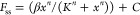
2where *x* is the cym (mM) or van (mM) inducer concentration.

### Statistical Analysis

All statistical analysis was done in R. Toggle switch performance metrics, and growth metrics were unit-scaled prior to downstream analysis. Hierarchical clustering was done using “hclust” function (distance = “Euclidean”, method = “complete”) from the base stats (v.3.6.2) package. Principal component analysis and procrustes superimposition analysis were done using the Vegan (v.2.6–4) package with functions “rda” and “protest” respectively. The *M*^2^ statistic from Procrustes Superimposition analysis (scale = TRUE, symmetric = TRUE) was tested for significance by a permutation approach (n = Inf, maximum number of iterations). Briefly, observations in one matrix are randomly reordered while maintaining the covariance structure within the matrix, and a test statistic is calculated and recorded enough times to obtain a sizable null distribution. A *P-*value for each statistic is then calculated, representing the probability of obtaining a statistic with a value equal to or more extreme of the experimental value.

## Data Availability

Experimental data files and R MarkDown scripts used for analysis and plotting of figures are publicly available online on the Open Science Framework database as part of the project name *Chan.RBS.Host.Context* (https://osf.io/4ye9w/).

## Supporting Information Available

Auxiliary data (Figures S1–S4); strain and DNA sequence metadata (Tables S1 and S2); and an overview of the specific protocol used to run DNA-BOT facilitated assembly of the pVCS plasmids (PDF)
