# Supplementary material for: Fine-Tuning Genetic Circuits via Host Context and RBS Modulation
Source: ACS Synth Biol. 2025 Jan 4;14(1):193–205. doi: 10.1021/acssynbio.4c00551 (PMC11744933; doi:10.1021/acssynbio.4c00551)
Supplement: Supplementary file 1 — sb4c00551_si_001.pdf [file sb4c00551_si_001.pdf]

Supporting Information

Fine-Tuning Genetic Circuits via Host Context and RBS Modulation

Dennis Tin Chat Chan[1], Lena Winter[1], Johan Bjerg[1], Stina Krsmanovic[1], Geoff S. Baldwin[3,4], Hans C. Bernstein[1,2]*

[1]Faculty of Biosciences, Fisheries and Economics, UiT - The Arctic University of Norway, 9019, Tromsø, Norway

[2]The Arctic Centre for Sustainable Energy, UiT - The Arctic University of Norway, 9019, Tromsø, Norway

[3]Department of Life Sciences, Imperial College London, South Kensington, London SW7 2AZ, UK

[4]Imperial College Centre for Synthetic Biology, Imperial College London, South Kensington, London SW7 2AZ, UK

* Correspondence: Hans. C. Bernstein (hans.c.bernstein@uit.no)

Keywords:

Synthetic Biology, Biodesign, Chassis-Effect, Genetic Circuit, Context-Dependence, Broad-Host-Range.

Supplementary Figures

Supplementary Figure S1

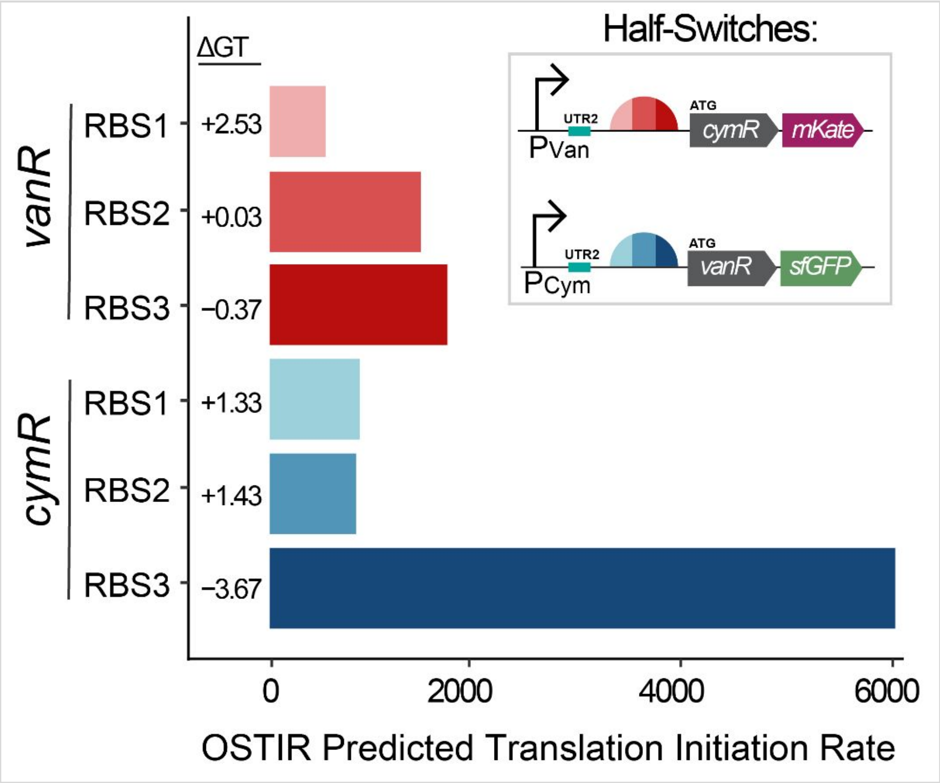

Supplementary Figure S1. Estimated translation initiation rate is context dependent. Inferred translation initiation rate of each RBS context regulating for *cymR* and *vanR* genes within the toggle switches by Open Source Translation Initiation Rates (OSTIR). ΔGT: change in Gibb's free energy associated with ribosome binding to the RBS part.

Supplementary Figure S2

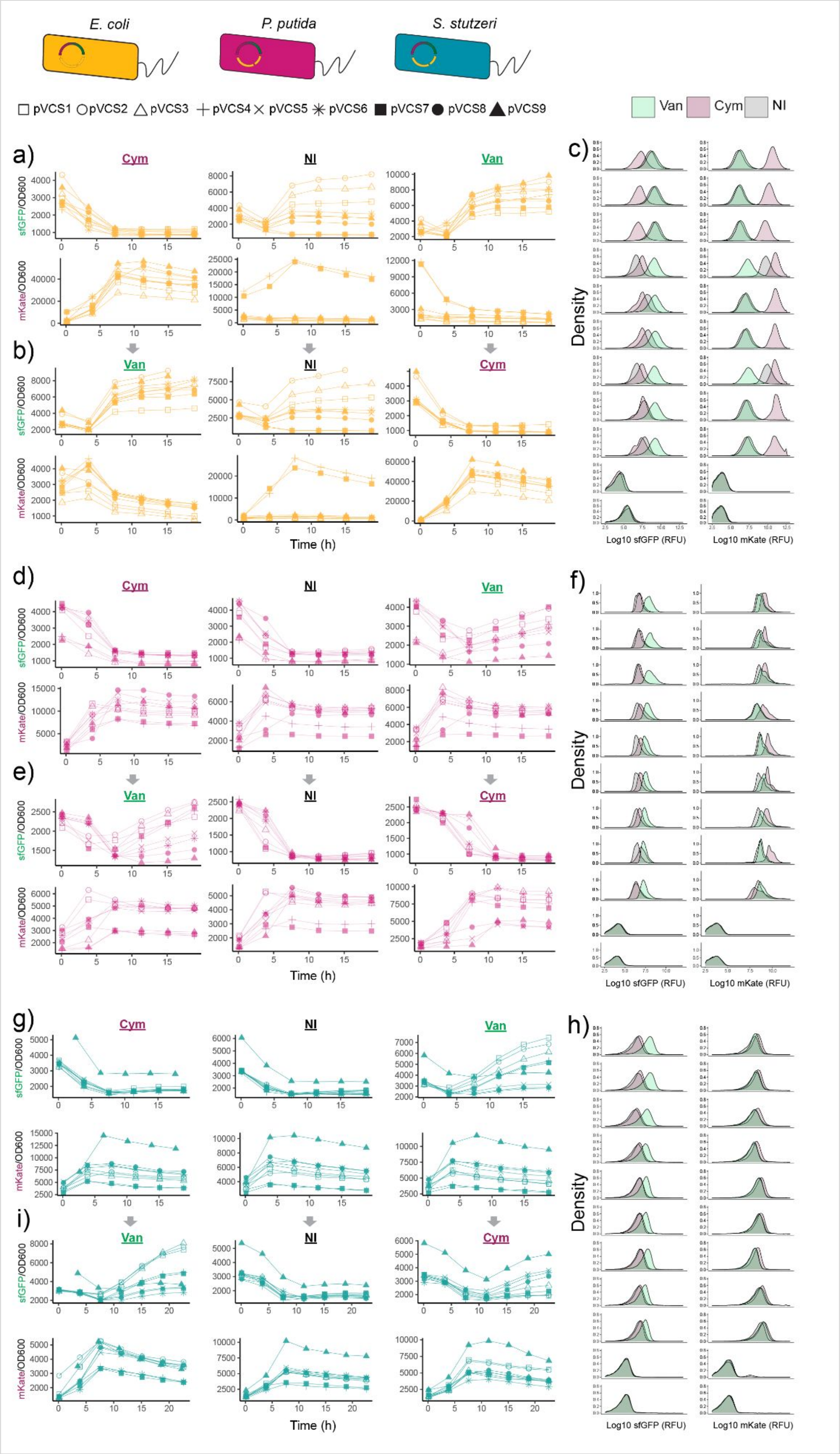

Supplementary Figure S2. Chassis-effect revealed through toggle switch fluorescence dynamics. Normalized fluorescence dynamics of toggled cells and fluorescence intensity distribution of late-phase cells for a-c) *Escherichia coli* DH5α, d-e) *Pseudomonas putida* KT2440 and g-i) *Stutzerimonas stutzeri* CCUG 11256. Cym: cumate; van: vanillate; NI: no inducer.

Supplementary Figure S3

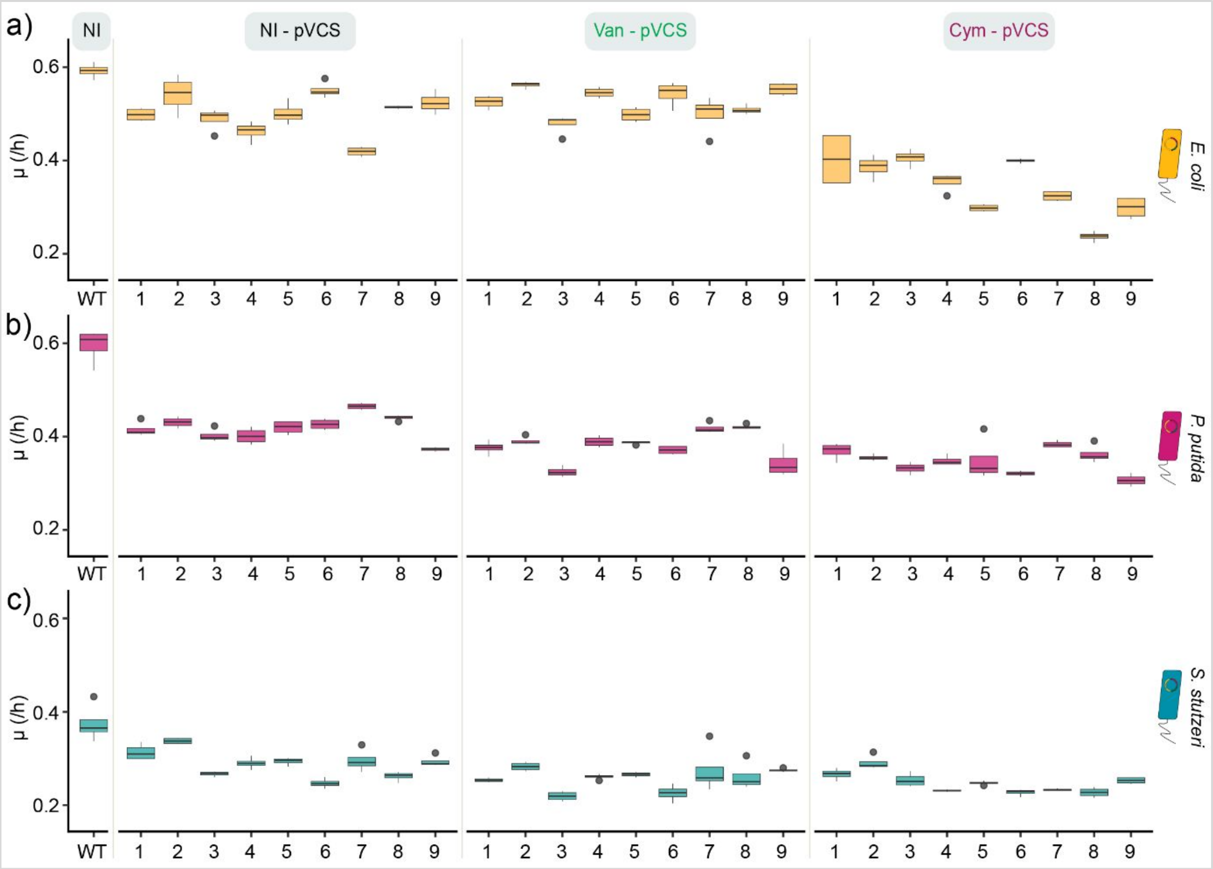

Supplementary Figure S3. Growth rates across genotype and induction states. Specific growth rates, used to determine growth burden, of WT and pVCS-bearing strains across non-induced (NI), van-induced (Van) and cym-induced (Cym) for a) *E. coli*, b) *P. putida* and c) *S. stutzeri*. Error bars show standard deviation, n = 4.

Supplementary Figure S4

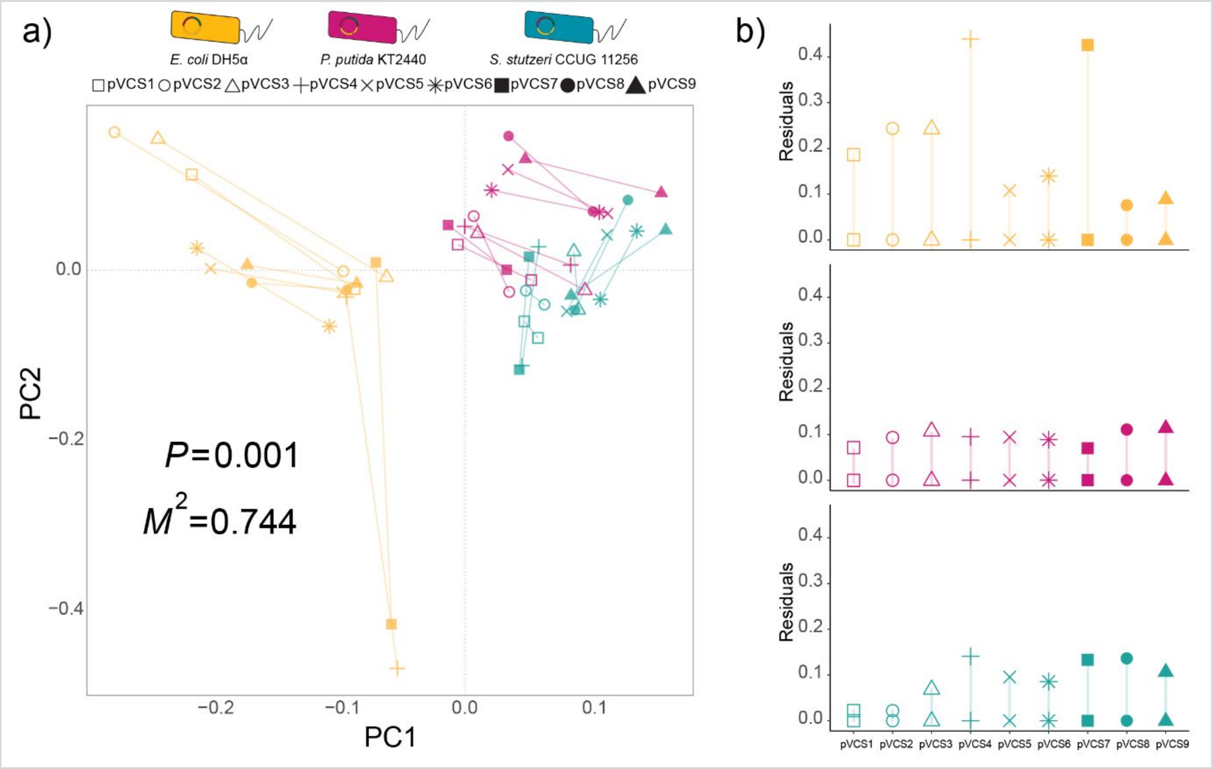

Supplementary Figure S4. Differences in growth between toggle switch variants significantly correlated with differences in toggle switch performance. a) PCA-based Procrustes Superimposition analysis of differential growth dynamics and differential toggle switch performance (toggle assay and induction metrics). Connecting lines indicate vector residuals. *P*: p-value. M^2: Gower statistic, the sum of squared vector residuals. b) Bar plot of residual vector between points.

Supplementary Tables

Supplementary Table S1

Supplementary Table 1. Species used in this study.

Name	Culture Collection Acc.	NCBI Assembly Acc.	Genotype	Reference
Escherichia coli DH5α	DSM 6897	GCF_002899475.1	WT	[1]
Pseudomonas putida KT2440	DSM 6125	GCF_000007565.2	WT	[2]
Stutzerimonas stutzeri CCUG11256	CCUG11256	GCF_000219605.1	WT	[3]
Escherichia coli DH5α	DSM 6897	NA	pVCS1	This Study
Escherichia coli DH5α	DSM 6897	NA	pVCS2	This Study
Escherichia coli DH5α	DSM 6897	NA	pVCS3	This Study
Escherichia coli DH5α	DSM 6897	NA	pVCS4	This Study
Escherichia coli DH5α	DSM 6897	NA	pVCS5	This Study
Escherichia coli DH5α	DSM 6897	NA	pVCS6	This Study
Escherichia coli DH5α	DSM 6897	NA	pVCS7	This Study
Escherichia coli DH5α	DSM 6897	NA	pVCS8	This Study
Escherichia coli DH5α	DSM 6897	NA	pVCS9	This Study
Pseudomonas putida KT2440	DSM 6125	NA	pVCS1	This Study
Pseudomonas putida KT2440	DSM 6125	NA	pVCS2	This Study
Pseudomonas putida KT2440	DSM 6125	NA	pVCS3	This Study
Pseudomonas putida KT2440	DSM 6125	NA	pVCS4	This Study
Pseudomonas putida KT2440	DSM 6125	NA	pVCS5	This Study
Pseudomonas putida KT2440	DSM 6125	NA	pVCS6	This Study
Pseudomonas putida KT2440	DSM 6125	NA	pVCS7	This Study
Pseudomonas putida KT2440	DSM 6125	NA	pVCS8	This Study
Pseudomonas putida KT2440	DSM 6125	NA	pVCS9	This Study
Stutzerimonas stutzeri CCUG11256	CCUG11256	NA	pVCS1	This Study
Stutzerimonas stutzeri CCUG11256	CCUG11256	NA	pVCS2	This Study
Stutzerimonas stutzeri CCUG11256	CCUG11256	NA	pVCS3	This Study
Stutzerimonas stutzeri CCUG11256	CCUG11256	NA	pVCS4	This Study
Stutzerimonas stutzeri CCUG11256	CCUG11256	NA	pVCS5	This Study
Stutzerimonas stutzeri CCUG11256	CCUG11256	NA	pVCS6	This Study
Stutzerimonas stutzeri CCUG11256	CCUG11256	NA	pVCS7	This Study
Stutzerimonas stutzeri CCUG11256	CCUG11256	NA	pVCS8	This Study
Stutzerimonas stutzeri CCUG11256	CCUG11256	NA	pVCS9	This Study

Supplementary Table S2

Supplementary Table 2. DNA sequences of parts used in this study.

Part	Sequence
PCym	CTCGGTACCAAATTCCAGAAAAGAGACGCTTTCGAGCGTCTTTTTTCGTTTTGGTCCGTGCCTACTC<br>TGGAAAATCTAACAAACAGACAATCTGGTCTGTTTGTATTATGGAAAATTTTTCTGTATAATAGAT<br>TCAACAAACAGACAATCTGGTCTGTTTGTATTATAGCGCTCAACGGGTGTGCTTCCCGTTCTGATG<br>AGTCCGTGAGGACGAAAGCGCCTCTACAAATAATTTTGTTTAA
PVan	GACGAACAATAAGGCCTCCCTAACGGGGGGCCTTTTTTATTGATAACAAAAGTGCCTACTCTGGAA<br>AATCTATTGGATCCAATTGACAGCTAGCTCAGTCCTAGGTACCATTGGATCCAATAGTAGTCACCG<br>GCTGTGCTTGCCGGTCTGATGAGCCTGTGAAGGCGAAACTACCTCTACAAATAATTTTGTTTAA
cymR	ATGAGCCCGAAACGTCGTACCCAGGCAGAACGTGCAATGGAAACCCAGGGTAAACTGATTGCAGC<br>AGCACTGGGTGTTCTGCGTGAAAAAGGTTATGCAGGTTTTCGTATTGCAGATGTTCCGGGTGCAGC<br>CGGTGTTAGCCGTGGTGCACAGAGCCATCATTTTCCGACCAAACTGGAACTGCTGCTGGCAACCTT<br>TGAATGGCTGTATGAGCAGATTACCGAACGTAGCCGTGCACGTCTGGCAAAACTGAAACCGGAAG<br>ATGATGTTATTCAGCAGATGCTGGATGATGCAGCAGAATTTTTTCTGGATGATGATTTTAGCATCG<br>GCCTGGATCTGATTGTTGCAGCAGATCGTGATCCGGCACTGCGTGAAGGTATTCAGCGTACCGTTG<br>AACGTAATCGTTTTGTTGTTGAAGATATGTGGCTGGGTGTGCTGGTGAGCCGTGGTCTGAGCCGTG<br>ATGATGCCGAAGATATTCTGTGGCTGATTTTTAACAGCGTTCGTGGTCTGGTAGTTCGTAGCCTGT<br>GGCAGAAAGATAAAGAACGTTTTGAACGTGTGCGTAATAGCACCCTGGAAATTGCACGTGAACGT<br>TATGCAAAATTCAAACGTTGA
vanR	ATGGACATGCCTCGTATTAAACCGGGTCAGCGTGTTATGATGGCACTGCGTAAAATGATTGCAAGC<br>GGTGAAATCAAAAGTGGTGAACGTATTGCAGAAATTCCGACCGCAGCAGCACTGGGTGTTAGCCG<br>TATGCCGGTTCGTATCGCACTGCGTTCACTGGAACAAGAAGGTCTGGTTGTTCGTCTGGGTGCACG<br>TGGTTATGCAGCCCGTGGTGTTAGCAGCGATCAGATTCGTGATGCAATTGAAGTTCGTGGTGTTCT<br>GGAAGGTTTTGCAGCACGTCGTCTGGCAGAACGTGGTATGACCGCAGAAACCCATGCACGTTTTGT<br>TGTACTGATTGCAGAAGGTGAAGCACTGTTTGCAGCCGGTCGCCTGAATGGTGAAGATCTGGATC<br>GTTATGCCGCATATAATCAGGCATTTCATGATACCCTGGTTAGCGCAGCAGGTAATGGTGCAGTTG<br>AAAGCGCACTGGCACGTAATGGTTTTGAACCGTTTGCAGCAGCCGGTGCACTGGCCCTGGATCTGA<br>TGGACCTGTCTGCCGAATATGAACATCTGCTGGCAGCACATCGTCAGCATCAGGCAGTTCTGGATG<br>CAGTTAGCTGTGGTGATGCCGAAGGTGCAGAACGTATTATGCGTGATCATGCACTGGCAGCAATTC<br>GTAATGCAAAAGTTTTTGAAGCAGCAGCAAGCGCAGGCGCACCGCTGGGTGCAGCATGGTCAATT<br>CGTGCAGATTGA
sfGFP	ATGCGTAAAGGCGAAGAGCTGTTCACTGGTGTCGTCCCTATTCTGGTGGAACTGGATGGTGATGTC<br>AACGGTCATAAGTTTTCCGTGCGTGGCGAGGGTGAAGGTGACGCAACTAATGGTAAACTGACGCT<br>GAAGTTCATCTGTACTACTGGTAAACTGCCGGTACCTTGGCCGACTCTGGTAACGACGCTGACTTA<br>TGGTGTTCAGTGCTTTGCTCGTTATCCGGACCATATGAAGCAGCATGACTTCTTCAAGTCCGCCAT<br>GCCGGAAGGCTATGTGCAGGAACGCACGATTTCCTTTAAGGATGACGGCACGTACAAAACGCGTG<br>CGGAAGTGAAATTTGAAGGCGATACTCTGGTAAACCGCATTGAGCTGAAAGGCATTGACTTTAAA<br>GAAGACGGCAATATCCTGGGCCATAAGCTGGAATACAATTTTAACAGCCACAATGTTTACATCAC<br>CGCCGATAAACAAAAAAATGGCACTAAAGCGAATTTTAAAATTCGCCACAACGTGGAGGATGGCA<br>GCGTGCAGCTGGCTGATCACTACCAGCAAAACACTCCAATCGGTGATGGTCCTGTTCTGCTGCCAG<br>ACAATCACTATCTGAGCACGCAAAGCGTTCTGTCTAAAGATCCGAACGAGAAACGCGATCATATG<br>GTTCTGCTGGAGTTCGTAACCGCAGCGGGCATCACGCATGGTATGGATGAACTGTAC
mKate	ATGTCAGAATTAATTAAAGAAAATATGCACATGAAATTATATATGGAAGGTACTGTCAACAATCA<br>TCATTTCAAATGCACATCCGAAGGTGAAGGTAAACCATATGAAGGCACACAAACAATGCGCATCA<br>AAGCAGTTGAAGGTGGACCCCTGCCCTTTGCGTTTGACATTCTCGCAACGAGCTTTATGTACGGGT<br>CTAAAACTTTTATCAATCACACCCAAGGCATTCCTGACTTTTTTAAACAGTCCTTTCCTGAAGGCTT<br>TACCTGGGAACGTGTAACAACTTATGAAGATGGCGGTGTACTTACAGCAACTCAAGATACGAGTT<br>TACAAGATGGCTGTCTGATTTACAATGTTAAAATCCGTGGCGTAAATTTCCCGAGTAACGGACCCG<br>TAATGCAAAAAAAAACTCTTGGTTGGGAAGCATCAACAGAAACCTTATATCCTGCGGACGGTGGC<br>TTAGAAGGACGCGCAGACATGGCACTGAAATTAGTTGGAGGCGGTCATTTAATCTGCAACCTGAA<br>AACAACCTATCGTTCCAAAAAACCCGCTAAAAACCTTAAAATGCCTGGAGTATACTATGTTGATCG<br>TCGCTTAGAACGTATTAAAGAAGCTGATAAAGAAACCTACGTTGAACAACATGAAGTAGCCGTAG<br>CCCGTTATTGTGACCTTCCGTCGAAATTAGGACATCGTTGA
UTR1	TTGAACACCGTCTCAGGTAAGTATCAGTTGTA
UTR2	TGTTACTATTGGCTGAGATAAGGGTAGCAGAA
UTR3	GTATCTCGTGGTCTGACGGTAAAATCTATTGT
RBS1	ATCACACAGGACTA
RBS2	AAAGAGGGGAAATA
RBS3	AAAGAGGAGAAATA

Supplementary Material

Supplementary Material S1

DNA-BOT Application Protocol Description
DNA-BOT was used to generate the four scripts to program the OpenTrons2 pipetting robot to assemble our toggle switches via BASIC DNA assembly. DNA-BOT application was run with default settings and the protocol was followed as described by Storch et al. (2020)[4] with the following differences:

In "2_purification_ot2_APIv2.8.py" script, the following lines were replaced with:

**Line**	**Replaced syntax**
125	pipette.flow_rate.aspirate=PIPETTE_ASPIRATE_RATE
126	pipette.flow_rate.dispense=PIPETTE_DISPENSE_RATE

The following settings for labware ID and parameters were changed to the indicated value:

**Step 2 – Labware IDs**	**Value**
Opentrons magnetic module gen2	magnetic_module_gen2
Opentrons 4-in-1 tubes rack	opentrons_24_tuberack_eppendorf_1.5ml_safelock_snapcap
96 well rigid PCR plate (clip and transformation steps)	armadillo_96_wellplate_200ul_pcr_full_skirt
96 well rigid PCR plate (purification and assembly steps)	armadillo_96_wellplate_200ul_pcr_full_skirt
Agar plate (transformation step)	armadillo_96_wellplate_200ul_pcr_full_skirt
Reservoir plate 21 mL 12 channels	nest_12_reservoir_15ml
96 deep well plate 2 mL wells	thermoscientificnunc_96_wellplate_2000ul

**Step 3 - Parameters**	**Value**
Magnetic module height (mm)	11
Settling time (min)	5

References

1. Chen, J., Li, Y., Zhang, K. & Wang, H. Whole-Genome Sequence of Phage-Resistant Strain Escherichia coli DH5α. *Genome Announcements* **6**, 10.1128/genomea.00097-18 (2018).
2. Martin-Pascual, M. *et al.* A navigation guide of synthetic biology tools for Pseudomonas putida. *Biotechnology Advances* **49**, 107732 (2021).
3. Gomila, M., Mulet, M., García-Valdés, E. & Lalucat, J. Genome-Based Taxonomy of the Genus Stutzerimonas and Proposal of S. frequens sp. nov. and S. degradans sp. nov. and Emended Descriptions of S. perfectomarina and S. chloritidismutans. *Microorganisms* **10**, 1363 (2022).
4. Storch, M., Haines, M. C. & Baldwin, G. S. DNA-BOT: a low-cost, automated DNA assembly platform for synthetic biology. *Synthetic Biology* **5**, ysaa010 (2020).
